# Comparing the Impact of Laparoscopic Sleeve Gastrectomy and Gastric Cancer Surgery on Resting-State Brain Activity and Functional Connectivity

**DOI:** 10.3389/fnins.2020.614092

**Published:** 2020-11-26

**Authors:** Yong Gu, Guanya Li, Jia Wang, Karen M. von Deneen, Kaichun Wu, Yan Yang, Junjun She, Gang Ji, Yongzhan Nie, Guangbin Cui, Yi Zhang, Shuixiang He

**Affiliations:** ^1^Department of Gastroenterology, First Affiliated Hospital of Xi’an Jiaotong University, Xi’an, China; ^2^Digestive System Department, Shaanxi Provincial Crops Hospital, Chinese People’s Armed Police Forces, Xi’an, China; ^3^Center for Brain Imaging, School of Life Sciences and Technology, Xidian University, Xi’an, China; ^4^State Key Laboratory of Cancer Biology, National Clinical Research Center for Digestive Diseases and Xijing Hospital of Digestive Diseases, Air Force Medical University, Xi’an, China; ^5^Department of Radiology, Tangdu Hospital, Air Force Medical University, Xi’an, China

**Keywords:** laparoscopic sleeve surgery, gastrectomy cancer surgery, fMRI, resting-state, functional connectivity

## Abstract

Laparoscopic sleeve gastrectomy (LSG) is one of the most performed bariatric surgeries in clinical practice. Growing neuroimaging evidence shows that LSG induces brain functional and structural alterations accompany with sustained weight-loss. Meanwhile, for clinical treatment of gastric cancer, stomach removal surgery is a similar procedure to LSG. It is unclear if the gastric cancer surgery (GCS) would induce the similar alterations in brain functions and structures as LSG, and it would help to clarify the specificity of the LSG. We recruited 24 obese patients who received LSG in the LSG group and 16 normal weight patients with gastric cancer who received GCS as the control group. Functional magnetic resonance imaging was employed to investigate the differences and similarity of surgery’s impact on resting-state brain activity and functional connectivity (RSFC) between LSG and GCS groups. Both LSG and GCS groups showed increased activities in the posterior cingulate cortex (PCC) and supplementary motor area (SMA) as well as the decreased RSFC of PCC- dorsomedial prefrontal cortex and SMA- dorsolateral prefrontal cortex. There were decreased resting-state activity of hippocampus and putamen in LSG group and increases in GCS group. In LSG group, resting-state activities of hippocampus and putamen were correlated with craving for high-caloric food and body mass index after surgery, respectively. These findings suggest LSG induced alterations in resting-state activity and RSFC of hippocampus and putamen specifically regulate the obese state and overeating behaviors in obese patients.

## Introduction

Bariatric surgery (BS) is the most effective treatment for morbid obesity and producing sustained weight-loss (Peterli et al., 2018). Laparoscopic sleeve gastrectomy (LSG) is one of the most performed procedures in clinical practice, and normally lead to weight loss between 20 and 35% of original weight (Peterli et al., 2018). Growing neuroimaging evidence indicates that LSG induces brain functional and structural alterations in regions and circuits implicated in reward (caudate, ventral tegmental area) (Faulconbridge et al., 2016; Zhang et al., 2016; Wang et al., 2020), emotion/memory [hippocampus (HIPP), amygdala (AMY)] (Zhang et al., 2019), self-referential processing [posterior cingulate cortex (PCC), precuneus] (Li et al., 2018; Liu et al., 2019), interoception (insula) (Wang et al., 2020) and inhibitory control [dorsolateral prefrontal cortex (DLPFC), anterior cingulate cortex (ACC)] (Li et al., 2019; Hu et al., 2020), which those changes tend to be levels of normal weight subjects; and highlight their critical role playing in the long-term weight loss post-surgery (Behary and Miras, 2015; Lin and Qu, 2020). Meanwhile, for clinical treatment of gastrointestinal disease, there are other surgical procedures (i.e., stomach removal surgery for treating gastric cancer) which are similar to LSG by removing partial of the stomach (Kim et al., 2017; Omori et al., 2020). It is not clear if the gastric cancer surgery (GCS) would induce the similar alterations in brain functions and structures as LSG, and it would help to clarify the specificity of the LSG.

Neuroimaging studies on obese individuals have shown the hyperactivations of striatum and limbic regions in responses to food-cues were associated with higher food craving than normal weight subjects, and structural abnormalities in prefrontal cortex (PFC) and caudate were associated with body mass index (BMI) and food addiction (Stoeckel et al., 2008; Pursey et al., 2014; Zhang et al., 2016). Overeating in obesity have been strongly attributed to the imbalance between the reward and inhibitory control circuits which is a consequence of conditioned learning and the resetting of reward thresholds following the long-term consumption of large quantities of high-calorie foods (Volkow et al., 2011). Previous studies related to LSG showed changes in functions (i.e., activity) and structures (i.e., volume) in those above regions were associated with the weight-loss and the reduction in food craving after surgery (Faulconbridge et al., 2016; Li et al., 2019; Wang et al., 2020). Recovery of brain abnormalities following surgery can be also recognized as the result of the conditioned learning of the restrictive feeding and the resetting of satiety induced reward (Duan et al., 2020). Therefore, LSG induced alterations in brain functions specifically regulate the obese state and overeating behaviors in obese patients. However, for patients with gastric cancer, they usually have normal weight and normal eating behavior; GCS induced changes in regions involved in food reward processing might different from LSG. Both LSG and GCS involve removing part of the stomach and changing the structure of gastrointestinal tract which has directly impact on the interaction between the gastrointestinal and central nervous system, and these two surgical procedures might have similar impact on brain.

In addition, previous studies on BS just compared the brain differences between pre- and post-surgery and were limited by the lack of a control group (Faulconbridge et al., 2016). Here, we recruited normal weight patients with gastric cancer who would receive GCS as the control group, and employed resting-state functional magnetic resonance imaging (RS-fMRI) to investigate the differences and similarity of surgery’s impact on resting-state brain activity and functional connectivity compared with LSG. We hypothesized that LSG would induce different brain alterations in striatum and limbic regions involved with food reward processing. With regards to the similar procedure of LSG and GCS, both LSG and GCS would also have modulation on brain function.

## Materials and Methods

### Subjects

Twenty-nine obese subjects (BMI ≥ 30) and twenty-four patients with gastric cancer were recruited for LSG and GCS surgeries at Xijing Gastrointestinal Hospital affiliated to the Air Force Medical University in Xi’an, China. Obese individuals with psychiatric/neurological diseases, previous intestinal surgery/disease, organ dysfunction or waist circumference (WC) > the interior diameter of the MRI scanner were excluded (Meng et al., 2018). Two obese candidates were disqualified (one had a WC > the interior diameter of the scanner and one withdrew). Three obese subjects withdrew after LSG surgery due to long distance travel. A total of 24 obese patients (age: 25.63 ± 1.59 years, sex: 9 male/15 female, BMI: 40.24 ± 1.01 Kg/m^2^, Table 1) remained in the LSG group and completed the pre-surgical MRI scan (PreLSG) and the same MRI scans 1-month post-surgery (PostLSG). Five patients with gastric cancer who have a history of alcohol addiction and three patients with cerebral infarct were excluded. Sixteen patients with gastric cancer (age: 56.56 ± 1.82 years, sex: 7 male/9 female, BMI: 20.91 ± 0.46 Kg/m^2^, Table 1) underwent MRI scan before (PreGCS) and 1 month after (PostGCS) gastric cancer surgery. The experimental protocol was approved by the Institutional Review Board of Xijing Hospital. The experiments were conducted in accordance with the Helsinki Declaration of 1975 (as revised in 1983). All participants were informed of the nature of the research and provided written informed consent.

**TABLE 1:** Demographic and clinical information of LSG and GCS groups.

	PreLSG (24)(Mean ± SE)	PostLSG (24)(Mean ± SE)	PreGCS (16)(Mean ± SE)	PostGCS (16)(Mean ± SE)	PreLSG vs. PreGCS	
	
					*T*	*P*
Age (years)	25.631.59	25.631.59	56.561.82	56.561.82	−12.59	<0.001
Sex	9M/15F	9M/15F	7M/9F	7M/9F	0.16	0.693
BMI (kg/m^2^)	40.241.01	35.711.07	20.910.46	19.890.47	14.96	<0.001
HiCal food craving	62.635.80	41.425.22	42.503.45	37.814.72	2.98	0.005
LoCal food craving	50.635.40	41.085.32	42.193.19	36.254.07	1.34	0.187
HAMD	11.421.55	8.831.17	11.561.54	10.312.02	−0.06	0.949
HAMA	12.421.84	11.081.30	13.002.13	12.002.51	−0.21	0.839

*BMI, body mass index; HAMD, Hamilton Depression Rating Scale; HAMA, Hamilton Anxiety Rating Scale; PreLSG, obese patients who received MRI scan before surgery; PostLSG, obese patients who received MRI scan at 1 month after surgery; PreGCS, patients with gastric cancer who received MRI scan before surgery; PostGCS, patients with gastric cancer who received MRI scan 1 month after the first scan; SE, standard error.*

### Experimental Design

All participants underwent 12-h overnight fasting, and MRI scans were performed between 9 and 10 AM. A designated clinician rated severity of subjects’ anxiety using Hamilton-Anxiety-Rating-Scale (Hamilton, 1959) and depression using Hamilton-Depression-Rating-Scale (Hamilton, 1960). The anxiety and depression measures were used to exclude subjects with a psychiatric disorder. Participants were instructed to rate their level of craving for high- (HiCal) and low-caloric (LoCal) food using a visual analog scale (range 0–100) (Ding et al., 2020; He et al., 2020). All clinical measurements were identically conducted before (baseline) and 1-month after surgery. Two sample *t*-tests were used to examine the differences between LSG and GCS groups before surgery. Two-way repeated measures ANOVA was implemented in SPSS 22 to model the main and interaction effects on group and surgery on behavioral/clinical data. Paired *t*-tests were utilized as *post hoc* tests where ANOVA indicated a significant main/interaction effects.

### MRI Acquisition

The experiment was carried out using a 3.0T GE (Signa Excite HD, Milwaukee, WI, United States) scanner. First, a high-resolution structural image for each subject was acquired using three-dimensional magnetization-prepared rapid acquisition gradient-echo sequences with a voxel size of 1 mm^3^ and with an axial fast spoiled gradient-echo sequence (TR = 7.8 ms, TE = 3.0 ms, matrix size = 256 × 256, field of view = 256 mm × 256 mm, slice thickness = 1 mm and 166 slices). Then, resting-state functional images were acquired using a gradient-echo T2^∗^-weighted echo planar imaging sequence. For each subject, 180 axial volume scans were obtained with the following parameters: TR = 2,000 ms, TE = 30 ms, matrix size = 64 × 64, FOV = 256 mm × 256 mm, flip angle = 90 degrees, in-plane resolution of 4 mm^2^, slice thickness = 4 mm and 32 axial slices. The scan for RS-fMRI lasted 360 s. Subjects were instructed to open their eyes and watch the fixation during the entire scanning procedure.

### Image Processing

Imaging data were preprocessed using Statistical Parametric Mapping 12 (SPM12^1^). Specifically, the first 10 time points were removed to minimize non-equilibrium effects in fMRI signal, and then slice-timing, head movement correction, and spatial normalization (voxel size of 3 mm × 3 mm × 3 mm) were performed. There was no significant interaction/main effect of Group/Time on estimating subjects’ motion (*P* > 0.05) for mean/maximum frame-wise displacement calculated from 6 translation/rotation parameters which were obtained from the realignment process. Demeaning/detrending were performed and head-motion parameters, white-matter signals, cerebrospinal-fluid signals and global signals were regressed out as nuisance covariates. fMRI time points that were severely affected by motion were removed using a “scrubbing method” (FD value > 0.5 mm, and ΔBOLD of DVARS > 0.5%), and <5% of time points were removed per subject (Power et al., 2014). Finally, band-pass temporal filtering (0.01–0.08 Hz) was used to remove effects of very low-frequency drift/high-frequency noise using REST toolkit^2^.

### Amplitude of Low Frequency Fluctuations Analysis

Amplitude of low frequency fluctuations (ALFF) analysis was carried out using REST software (see text footnote 2). The preprocessed time series was first converted to the frequency domain with a fast Fourier transform and the power spectrum was obtained. The square root of the power spectrum was computed at each voxel and the averaged square root was obtained in the 0.01–0.08 Hz bandwidth at each voxel. For standardization, the ALFF of each voxel was further divided by the whole-brain mean ALFF values and *z*-score normalization was used to convert ALFF maps into normally distributed coefficient maps. Two-way repeated measures ANOVAs were performed to model the interaction effects of group and surgery and the main effect of surgery on ALFF. Age and sex were entered as covariates to control for differences between groups in these variables. Results were corrected for multiple comparisons using family wise error (FWE) correction at cluster-level correction approach (*P*_FWE_ < 0.05) with a minimum cluster size of 30 and a cluster-forming threshold of *P* < 0.001.

### Resting-State Functional Connectivity Analysis

The clusters with significant interaction or surgery effects were selected as the seed regions, then seed-region-based RSFC analyses were carried out. Mean time series of each seed region from the resting-state scan was extracted, and then strength of RSFC between each voxel and the seed region was estimated using Pearson correlation coefficient between average time-varying signal in the seed and voxel in the brain. Fisher z-transform was used to convert correlation maps into normally distributed coefficient maps. Two-way repeated measures ANOVAs were implemented to model the interaction effects and surgery effects on seed-based RSFC (*P*_FWE_ < 0.05, cluster size of 30, cluster-forming threshold of *P* < 0.001).

## Results

### Demographic Characteristics

At baseline, there were no significant differences in sex, craving for LoCal food, HAMD and HAMA between LSG and GCS groups (*P* > 0.05, Table 1). PreLSG group was younger (*t* = −12.56, *P* < 0.001) and had higher BMI (*t* = 14.96, *P* < 0.001) and craving for HiCal food (*t* = 2.98, *P* = 0.005) than PreGCS group. There were significant group × surgery interaction effects on BMI [*F*(1,38) = 83.151, *P* < 0.001] due to larger reduction in BMI in LSG group than GCS group (*t* = 9.12, *P* < 0.001). There were significantly decreased craving for HiCal food (*t* = −3.24, *P* = 0.004) and HAMD (*t* = −2.75, *P* = 0.011) in LSG group post-surgery.

### Altered ALFF/RSFC

**FIGURE 1:**
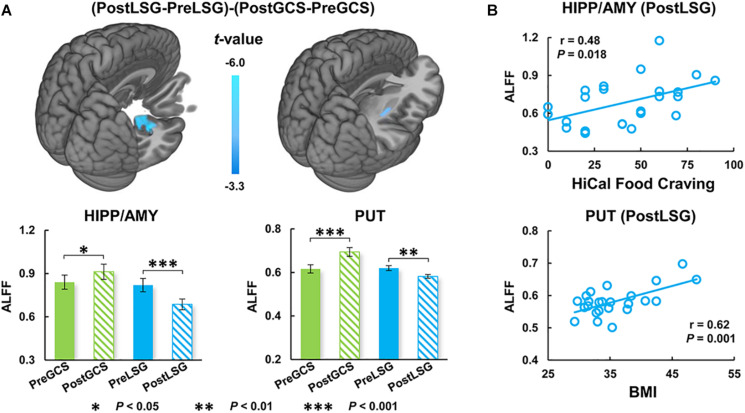
Interaction effects (group × surgery) for ALFF (cluster size-corrected, *P*_FWE_ < 0.05). **(A)** There were significant interaction effects (group × surgery) on ALFF in the HIPP/AMY and PUT. LSG group after surgery had decreased ALFF and GCS group had increased ALFF in HIPP/AMY and PUT. **(B)** Correlation analysis between behavioral measurements and ALFF. The error bars indicate the standard error. ALFF, Amplitude of low-frequency fluctuation; LSG, laparoscopic sleeve gastrectomy; GCS, gastric cancer surgery; HIPP, hippocampus; AMY, amygdala; PUT, Putamen.

**FIGURE 2:**
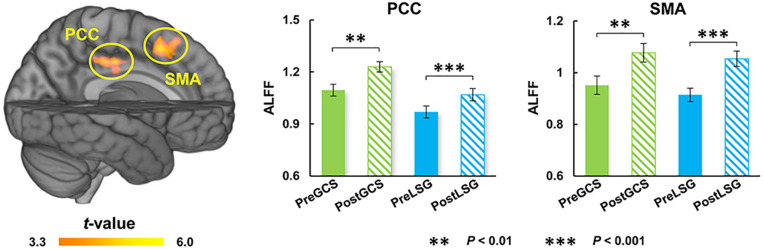
Surgery effects for ALFF (cluster size-corrected, *P*_FWE_ < 0.05). There were significant time effects on ALFF in the PCC and SMA. Both LSG and GCS group after surgery had increased ALFF in PCC and SMA. The error bars indicate the standard error. ALFF, Amplitude of low-frequency fluctuation; LSG, laparoscopic sleeve gastrectomy; GCS, gastric cancer surgery; PCC, posterior cingulate cortex; SMA, supplementary motor area.

**FIGURE 3:**
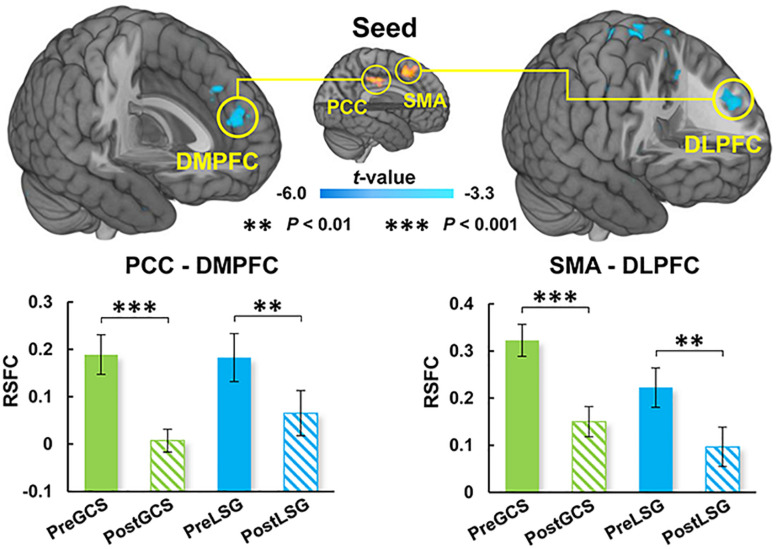
Surgery effects for RSFC (cluster size-corrected, *P*_FWE_ < 0.05). There were significant surgery effects on the RSFC of PCC-DMPFC and SMA-DLPFC. Both LSG and GCS group after surgery had decreased RSFC of PCC-DMPFC and SMA-DLPFC. The error bars indicate the standard error. RSFC, resting-state functional connectivity; PCC, posterior cingulate cortex; DMPFC, dorsomedial prefrontal cortex; SMA, supplementary motor area; DLPFC, dorsolateral prefrontal cortex.

There were significant interaction effects on resting-state activity (ALFF) in the HIPP and putamen (PUT) (Table 2). Specifically, LSG group showed decreased ALFF in HIPP (*t* = −7.04, *P* < 0.001) and PUT (*t* = −3.35, *P* = 0.003) (*P*_FWE_ < 0.05, Figure 1A). Conversely, GCS group showed significant decreased ALFF in HIPP (*t* = 2.91, *P* = 0.011) and PUT (*t* = 4.41, *P* < 0.001) (Figure 1A). In LSG group after surgery (PostLSG), HIPP activity was positively or negatively associated with craving for HiCal food (*r* = 0.48, *P* = 0.018, Figure 1B) and BMI was significantly positively or negatively correlated with PUT activity (*r* = 0.62, *P* = 0.001, Figure 1B). There were significant main effects on resting-state activity in PCC and supplementary motor area (SMA) (*P*_FWE_ < 0.05, Figure 2). Both LSG and GCS groups showed increased resting-state activity in PCC (LSG: *t* = 5.15, *P* < 0.001; GCS: *t* = 3.48, *P* = 0.003) and SMA (LSG: *t* = 5.52, *P* < 0.001; GCS: *t* = 3.40, *P* = 0.004) after surgery (Figure 2). There were significant surgery effects on RSFC between the PCC seed and the dorsomedial prefrontal cortex (DMPFC), and between the SMA seed and DLPFC (*P*_FWE_ < 0.05, Figure 3). *Post hoc* tests showed decreased RSFC of PCC-DMPFC (LSG: *t* = −3.09, *P* = 0.001; GCS: *t* = −4.37, *P* < 0.0031) and SMA-DLPFC (LSG: *t* = −3.08, *P* = 0.005; GCS: *t* = 4.39, *P* < 0.001) in both LSG and GCS groups (Figure 3). There were no significant FC changes regarding HIPP/AMY and PUT.

**TABLE 2:** Interaction effects (group × surgery) and surgery effects for ALFF (cluster size-corrected, *P*_FWE_ < 0.05).

Regions	Brodmann area(s)	Cluster size	Peak coordinates			Peak *t*-value
	
			*X*	*Y*	*Z*	
**Interaction effects:**						
HIPP/AMY	28, 34, 35	131	18	−3	−18	−5.54
PUT		57	30	−6	6	−5.13
**Surgery effects:**						
PCC	23	64	−6	−21	39	6.77
SMA	6	124	−6	15	51	5.50

*HIPP, hippocampus; AMY, amygdala; PUT, Putamen; PCC, posterior cingulate cortex; SMA, supplementary motor area.*

## Discussion

In the current study, we employed RS-fMRI to examine the differences in brain activity and functional connectivity between LSG and GCS. We found that both LSG and GCS groups showed increased PCC and SMA activity as well as the decreased RSFC of PCC-DMPFC and SMA-DLPFC. There were decreased resting-state activity of HIPP and PUT in LSG group and increases in GCS group. In LSG group, resting-state activities of HIPP and PUT were correlated with craving for HiCal food and BMI after surgery, respectively.

### Different Impact on ALFF Between LSG and GCS

In obesity, evidence indicates that HIPP has great impact on food-intake through learning and memory mechanisms (Weilbächer and Gluth, 2017). HIPP integrates external sensory information (visuospatial and olfactory) with the internal context (endocrine, gustatory, and gastrointestinal interoceptive stimuli) to make the decision to eat and how much and when to eat (Davidson et al., 2007; Kanoski and Grill, 2017). Recent study reported that HIPP showed both stronger resting-state activity and hyperactivation in responses to HiCal food-cues in obese individuals, and further mediation analysis reflected that basal dysfunctions in HIPP impact its regional responses to food-cues and eating behavior including food craving (Li et al., 2020). Obese subjects also showed lower gray matter density and higher response to HiCal food cues in PUT which is associated with processing of reward and stimuli salience than lean individuals (Stoeckel et al., 2008). In addition, LSG promotes significant recovery of brain function and structure in HIPP and PUT accompany with improvement of eating behavior (Zhang et al., 2016, 2019). Our findings are consistent with those previous reports on BS showing decreased HIPP and PUT activity in LSG group after surgery (Frank et al., 2014; Zhang et al., 2019). The association between resting-state activity of HIPP and craving for HiCal food and the association between ALFF in PUT and BMI in obese patients after LSG also suggest that LSG induced brain functional recovery playing an important role in the long-term weight-loss after surgery. It is noteworthy that the GCS group had even higher resting-state activity of HIPP and PUT after surgery and showed the opposite changes as compared with LSG group, suggesting that the changes in HIPP and PUT after LSG were associated with both surgery and obese state. In addition, previous implantable gastric stimulation study showed activation of HIPP and PUT, presumably from downstream stimulation of the vagus nerve and solitary nucleus, which suggested a functional connection between HIPP/PUT and the gastrointestinal tract (stomach) (Wang et al., 2006). Although the LSG and GCS group showed distinct altered trend in the HIPP and PUT, changes in these regions might due to the surgical procedures which involve the removal of part of stomach.

### Similar Impact on ALFF and RSFC Between LSG and GCS

Our results also showed increased resting-state activity of PCC and SMA in both LSG and GCS groups. The SMA serves to detect, engage, and direct attention toward behaviorally relevant sensory stimuli and plays an important role in the executive control of motor behavior, including inhibition of responses, as well as updating motor plans in accordance with current requirements (Nachev et al., 2008). The PCC is one of several midline cortical regions implicated in self-referential processing and is most active while processing one’s internal mental status (Northoff et al., 2006). In previous studies related to BS, activity of PCC was altered by surgery no matter which surgical procedure was performed (Li et al., 2018; Zoon et al., 2018). In the current study, increases in resting-state activity of PCC and SMA in both LSG and GCS groups might reflect the changes in gastrointestinal tract structure. There were also altered RSFC of PCC-DMPFC and SMA-DLPFC. DLPFC was the core region in neuronal circuits involved in top-down control and was strongly linked to several aspects of impulse-control, such as inhibitory-control, executive-attention and emotion-regulation (Volkow et al., 2011). DMPFC is part of the anterior DMN which is most active when processing internal mental status, such as self-referential thinking/auto biographical memory, and during external unfocused attention (Northoff et al., 2006). Both obese subjects and gastric cancer patients who underwent stomach removal surgery showed improved frontal lobe function such as attention and executive function (Kim et al., 2017; Hu et al., 2020). The enhanced RSFC of PCC-DMPFC and SMA-DLPFC could contribute to the improvements in cognitive function that have been reported in LSG and GCS.

## Conclusion

The current study investigated the difference and similarity of surgery’s impact on resting-state brain activity and RSFC between LSG and GCS. Our result showed LSG and GCS simultaneously increased resting-state activity in PCC and SMA which might reflect the changes in gastrointestinal tract structure. There were opposite changes in HIPP and PUT activity in LSG and GCS groups, and resting-state activity of HIPP/PUT were correlated with craving for HiCal food and BMI, respectively, in LSG group after surgery, suggesting LSG induced alterations in hippocampus and putamen functions specifically regulate the obese state and overeating behaviors in obese patients.

## Data Availability Statement

The datasets presented in this article are not readily available because the concern over patient privacy. Requests to access the datasets should be directed to SH.

## Ethics Statement

The studies involving human participants were reviewed and approved by the Institutional Review Board of Xijing Hospital. The patients/participants provided their written informed consent to participate in this study.

## Author Contributions

SH, YZ, and GC: conceptualization and design. JW, KD, KW, YY, JS, GJ, and YN: data acquisition. YG and GL: data analysis and writing the manuscript. All authors contributed to manuscript revision and approved it for publication.

## Conflict of Interest

The authors declare that the research was conducted in the absence of any commercial or financial relationships that could be construed as a potential conflict of interest.

## Footnotes

1 https://www.fil.ion.ucl.ac.uk/spm/

2 http://resting-fmri.sourceforge.net
